# Supplementary material for: Real-time telemetry monitoring of oxygen in the central complex of freely-walking Gromphadorhina portentosa
Source: PLoS One. 2019 Nov 11;14(11):e0224932. doi: 10.1371/journal.pone.0224932 (PMC6844484; doi:10.1371/journal.pone.0224932)
Supplement: S1 File — (DOCX) [file pone.0224932.s001.docx]

**Real-time Telemetry Monitoring of Oxygen in the Central Complex of freely-walking *Gromphadorhina portentosa***

Pier Andrea Serra^1,4,5^*, Paola Arrigo^1^, Andrea Bacciu^1^, Daniele Zuncheddu^1^, Riccardo Deliperi^1^, Diego Antón Viana^1^, Patrizia Monti^1^, Maria Vittoria Varoni^2^, Maria Alessandra Sotgiu^3^, Pasquale Bandiera^3^, Gaia Rocchitta^1,5^

^1^Department of Medical, Surgical and Experimental Medicine, Medical School, University of Sassari, Sassari, Italy.

^2^Department of Veterinary Medicine, Medical School, University of Sassari, Sassari, Italy.

^3^Department of Biomedical Sciences, Medical School, University of Sassari, Sassari, Italy.

^4^Institute of Sciences of Food Production, Italian National Research Council, Sassari, Italy.

^5^Mediterranean Center for Disease Control, University of Sassari, Sassari, Italy.

*****Corresponding author

E-mail: paserra@uniss.it (PS)

**Supporting Information (SI)**

**Telemetric device and USB transceiver unit**

The electronic circuit of the miniaturized telemetric device (Fig 3D in the main manuscript and Fig A and B in SI) was built using surface mounted components and was comprised of three different parts: the amperometric module, the microcontroller and the transceiver modules. As illustrated in Fig A, the amperometric module was made by soldering a MCP6044, six resistors and two capacitors on a 26.5 mm x 15.2 mm dual-side PCB.


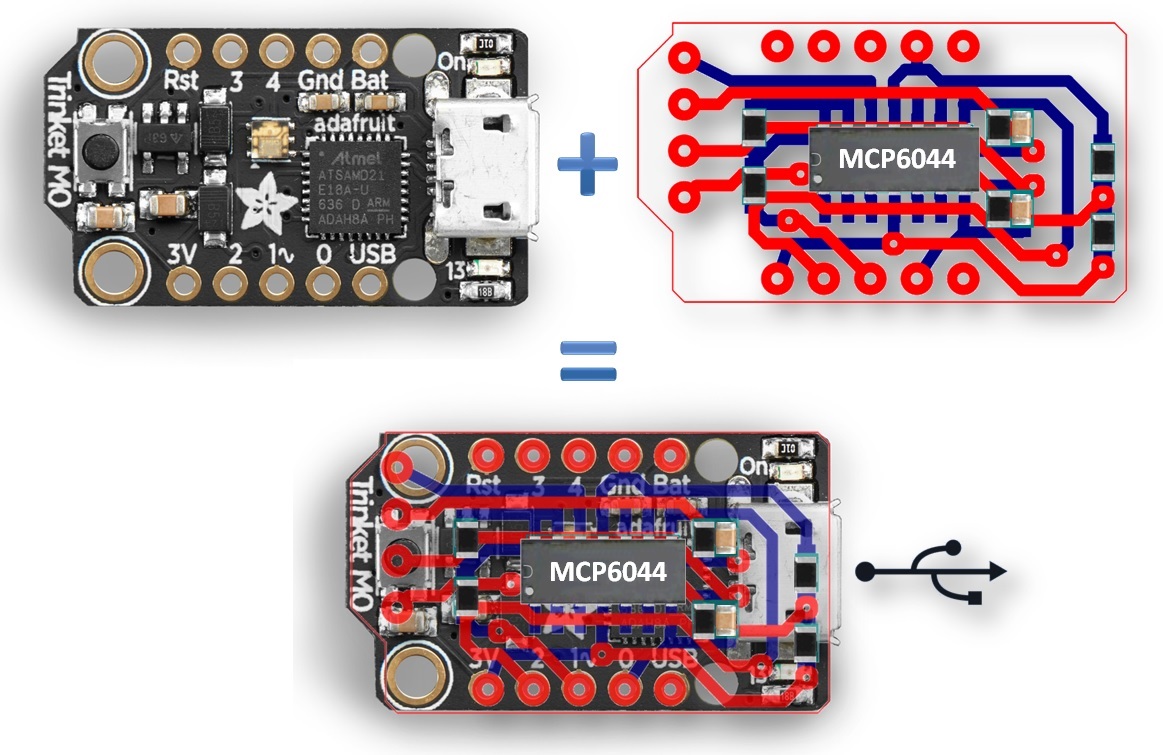


**Fig A. The amperometric and the microcontroller modules of the telemetric device.**

The MCP6044 has been designed for micropower applications consuming only 600 nA per OPA and can operate from a single-supply voltage with “railto-rail” inputs and outputs. The reference/auxiliary electrodes (RE/AE) are directly connected to the two-OPA potentiostatic circuit. The current-to-voltage (I/V) converters, are single-supply adaptations of a classical transimpedance amplifier. The transfer function of the I/V converters is:

${Vout}_{1,2}=-\left( I_{1,2} \cdot{Rf}_{1,2} \right)+Vapp$ (1)

in which I_1,2_ are the reduction current flowing through the WEs, Rf_1,2_ are the feedback resistors and Vapp is the potential applied to the WEs versus Vref (1.65V obtained through a resistive divider). Rf_1,2_ have two capacitors in parallel (Cf_1,2_) to complete the low pass filters with a cut-off frequency (Fcut-off) of 10 Hz. The values of Cf_1,2_ (1.5 nF) were calculated according to the equation:

${Cf}_{1,2}=\frac{1}{Fcut-off \cdot2\pi\cdot{Rf}_{1,2}}$ (2)

The Microchip ATSAMD21E18 is the heart of the digital module (Trinket M0). This is a 32-bit CMOS MCU based on a Cortex M0+ core with 256KB of Flash Memory and 32 KB of RAM. The microcontroller, working at 48 MHz, generated the 10-bit Vapp signal (DAC at pin 1~) and performed the A/D conversions of Vout_1,2_ using the internal 12-bit ADCs (at pin 0 and 2). Vapp signal was digitally subtracted from Vout_1,2_ and a serial data packet was generated and sent to the transceiver (Fig B, panel A).


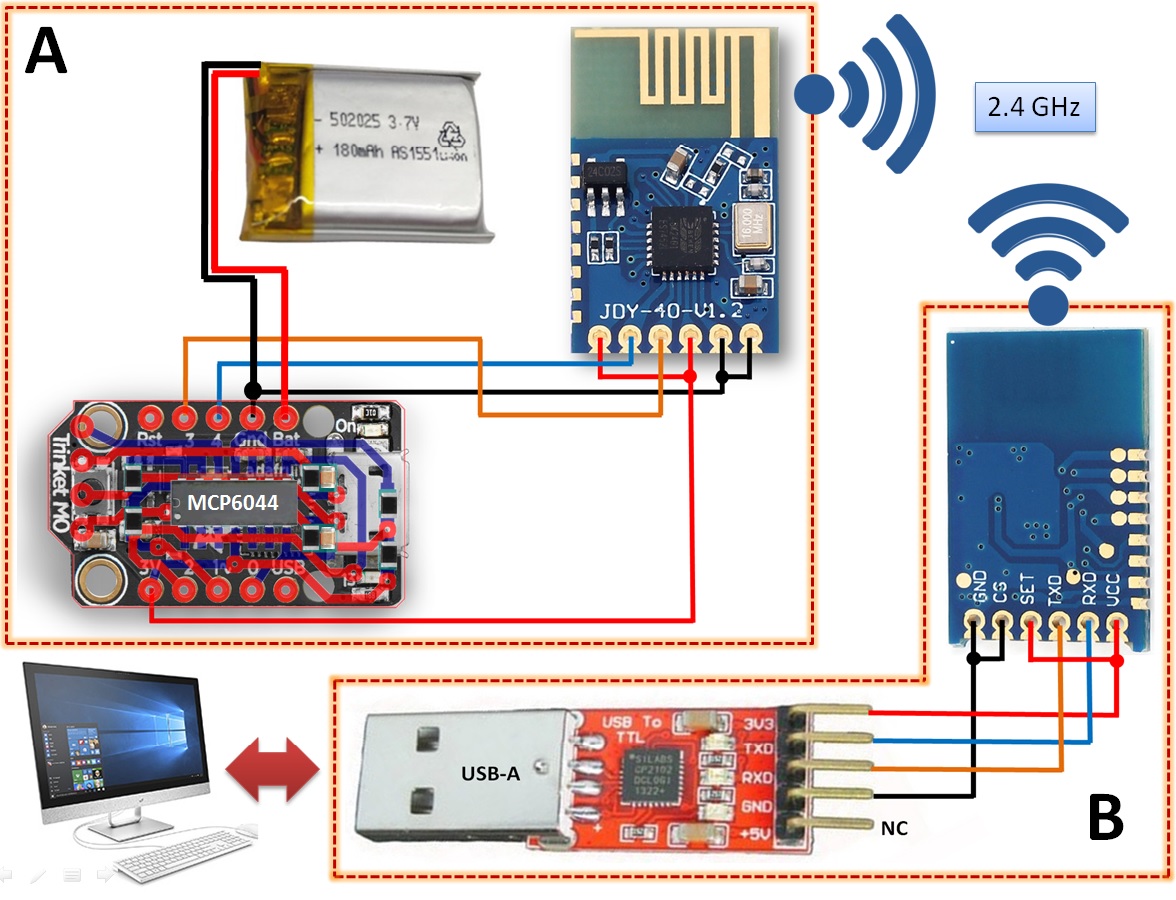


**Fig B. The transceiver modules wired to the telemetric device (A) and to the serial-to-USB converter (B).**

Two pins of the MCU were connected to the radio transceiver providing the TX and RX serial lines while the 3.3V power supply and GND signals were derived from the microcontroller module. The miniaturized transceiver (JDY-40) is an industrial serial module transmitting in the frequency of 2.4 GHz with internal antenna. In conjunction with the Trinket M0, this component allows the realization of a serial data transmitter working at the speed of 9600 baud. A 180 mAh, 3.7 V Li-Poly battery provided the power to the telemetric device for up to five hours of continuous transmission (5 Hz). The device was powered up simply by connecting its battery.

A second JDY-40 module was connected to a serial-to-USB converter and interfaced with a personal computer simulating a serial communication (COM) port (Fig B, panel B). Several JDY-40 modules can be paired by modifying their firmware for working with multiple devices at the same time (see JDY-40 datasheet).

**Firmware**

A USB micro socket in the Trinket M0 module provides the possibility of programming the MCU “on-board” in a few seconds by simply connecting it to the PC and saving the “code.py” file in the “CIRCUITPY” drive. This is possible thanks to a high-efficiency interpreter called CircuitPython freely downloadable from <https://circuitpython.org/board/trinket_m0/> (version 4.0.2). The firmware to drive the SAMD21 microcontroller was developed in Python language using Mu Editor, an open source integrated development environment, freely available at <https://codewith.mu/>. The program, which runs on the telemetry device, consists of a series of routines for the initialization and the setting up of the MCU (Fig C). The specialized code allows the UART initialization, the generation of the Vapp (*via* DAC) and a series of function for data conversion. One function in particular calculates the 16 Bit Cyclic Redundancy Check (CRC) of the serial string.


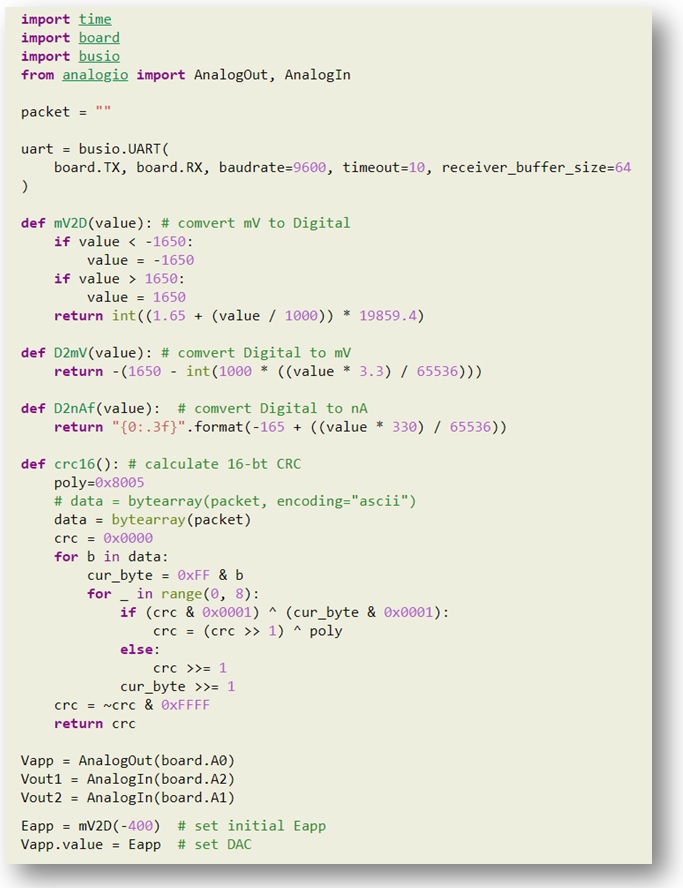


**Fig C. CircuitPython routines and initialization code for setting up the MCU.**

The core of the firmware consists in a infinite loop for continuous data acquisition and data transmission (Fig D).


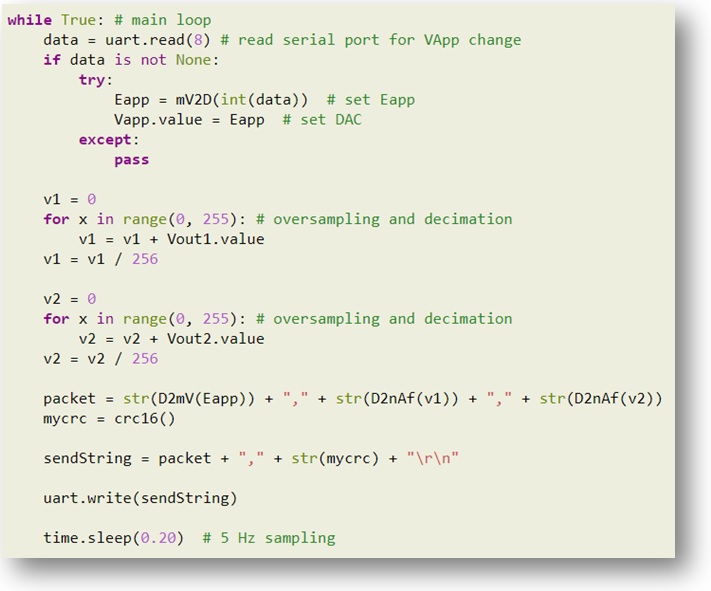


**Fig D. Main loop coded in CircuitPython and running in the MCU.**

The analogue signals Vout_1,2_ are digitized and sent to the transceiver module. The hardware ADC resolution (12 bit) was improved following the oversampling and averaging method:

$Fos= 4^{n} \cdot Fs$ (3)

where n is the number of additional bits of resolution (4), Fs is the sampling frequency (5 Hz) and Fos is the oversampling frequency (1280 Hz). In accordance with Nyquist’s theorem Fs was calculated as follows:

$Fs= 2 \cdot Fmax$ (4)

in which Fmax has been fixed to 2.5 Hz. To achieve this, the MCU acquired, accumulated and averaged 256 consecutive samples. This technique allowed to increase the theorical ADC resolution from 12 to 16 bits and significantly reduced the noise. The UART was constantly monitored for allowing the change of the Vapp in every moment and permitting the future use with oxidation sensors or first-generation oxidase biosensors.

**Software**

The software (NanoStat 2020), running on the PC, communicates with the USB transceiver unit by using the low-level CP2102 driver freely available from Silicon Laboratories Inc (<https://www.silabs.com/>). The serial routines and the graphic user interface were developed in Profilab Expert visual programming environment by Abacom (<https://www.electronic-software-shop.com>).


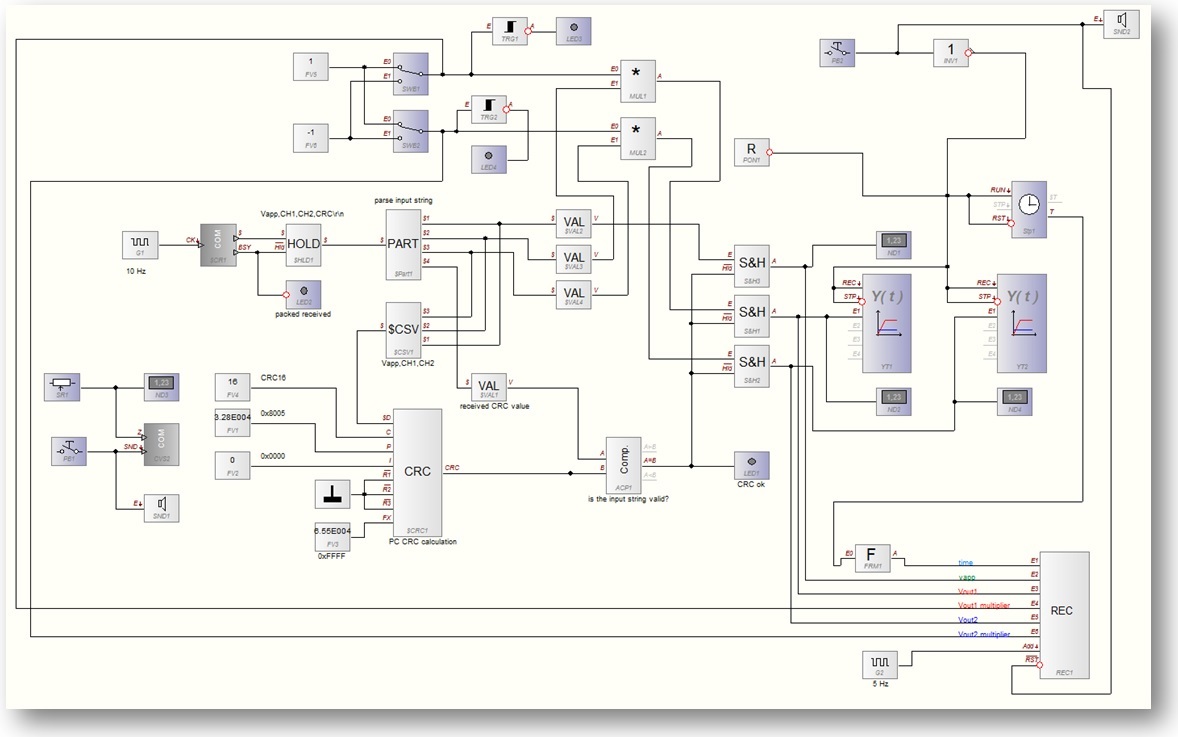


**Fig E. Visual representation of the software running in the PC.**

The communication starts with a serial polling (10 Hz) and ends with the validation of data received from the telemetric device (16-bir CRC). The application (capable of plotting, storing and retrieving data) can be interfaced the with the Local Area Network (LAN) and Internet via TCP/IP.


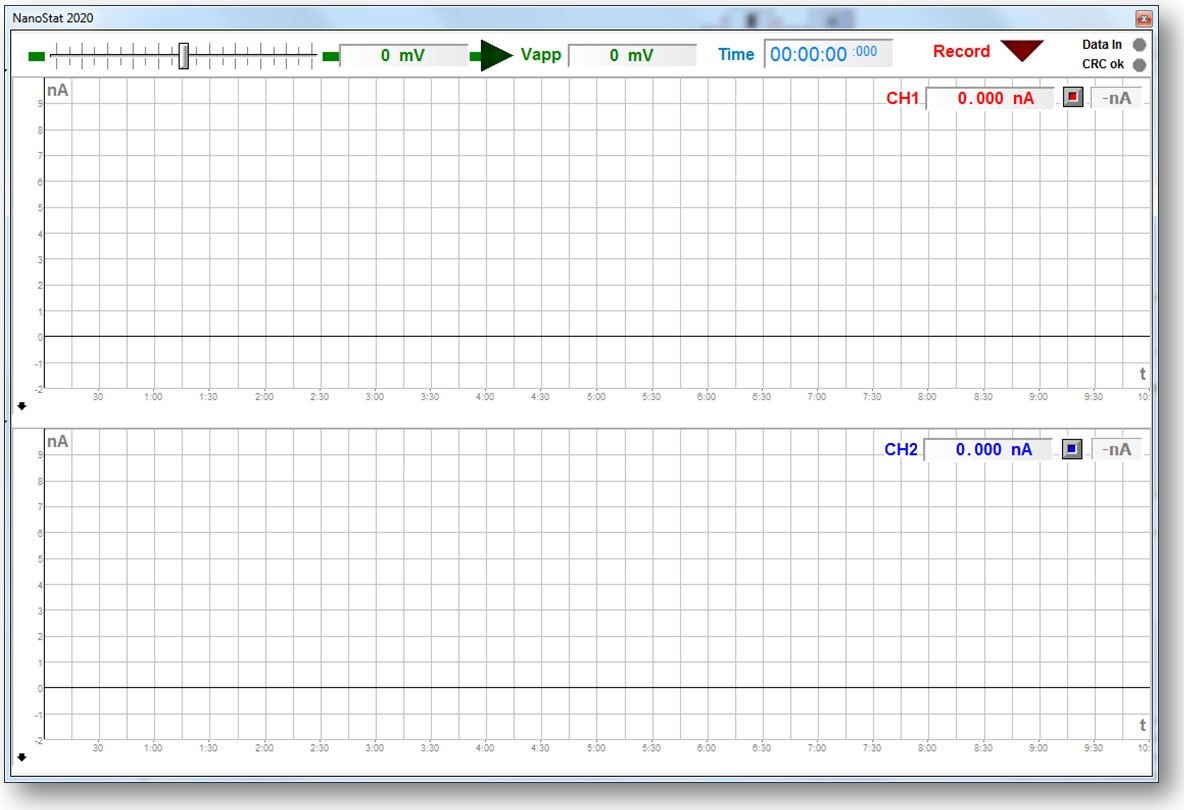


**Fig F. The two-channel Graphic User Interface (GUI) developed for monitoring oxygen signals in real-time.**

A software alarm was generated when the calculated CRC differed from the transmitted value or a data reception time-out occurred.

**Comparison among** **oxygen-detection telemetry systems**

At the state of the art only two telemetric systems for oxygen monitoring, with characteristics comparable to those proposed in this study, are present in literature. The first is the one developed by our research group which inspired the present study [1] while the second was developed by Russell and co-workers [2].

The table A summarizes the differences and similarities between the main parameters of the system proposed in this study compared with the devices described in the two studies cited above.

| **Parameter** | **Telemetric oxygen detection system** | | |
| --- | --- | --- | --- |
|  | **Bazzu et al., 2009 [1]** | **Russel at al., 2012 [2]** | **This study** |
| **Sensor material** | Epoxy-carbon | Carbon paste | Epoxy-carbon |
| **Sensor diameter** | 180 µm | 200 µm | 180 µm |
| **Sensor channels** | 1 | 1 | 2 |
| **Monitored Brain region** | Striatum  (rat) | Striatum  (rat) | Central Complex  (Gromphadorhina portentosa) |
| **Recording time** | 1 week | 3 weeks | 1 week |
| **Microcontroller**  **(MCU)** | PIC12F683  (Arizona Microchip) | nRF24LE1  (Nordic Semiconductor) | ATSAMD21E18  (Arizona Microchip) |
| **MCU speed** | 8 MHz | Not defined | 48 MHz |
| **ADC** | 10 bit  (14 bits with oversampling) | 12 bit | 12 bit  (16 bits with oversampling) |
| **DAC** | None | None | 10 bit |
| **Transmission Frequency** | 434 MHz | 2.4 GHz | 2.4 GHz |
| **Bidirectional telemetry** | No | Not defined | Yes |
| **Data transmission speed** | 2400 baud | 2 KHz | 9600 baud |
| **Battery** | Lithium coin battery (CR1216, 25 mAh) | Not defined | Lithium-ion-polymer (Li-Poly, 180 mAh) |
| **Rechargeable battery** | No | Yes | Yes |
| **Power consumption** | 3.25 mA  (transmission) | Not defined | 32 mA  (continuous) |
| **Hardware circuit** | Available | Not available | Available |
| **Telemetry system positioning** | Head-mounted  (external) | Implanted  (intra-abdominal) | Pronotum-mounted  (external) |
| **Firmware source** | Not available | Not available | Available |
| **Simple firmware update** | No | No | Yes |

**Table A. Comparison of the main parameters of the telemetric devices used for monitoring cerebral oxygen in freely-moving animals.**

Compared to the previous systems, the proposed device has undoubted advantages regarding the ADC, the DAC and the power of the MCU. The greatest limitation is represented by the high power consumption that can be optimized in the future thanks to the availability of new CircuitPython libraries. Russell's project [2], designed to be implanted in the rat peritoneum, is not optimal for use with *Gromphadorhina portentosa* where that type of implant cannot be accomplished. The availability of hardware schematics and firmware sources can allow other researchers to optimize the proposed system and to miniaturize it in the future.

**Stereotaxic frame adapter**

An adapter was developed with the aim of immobilizing the anesthetized animal's head and allowing the experimenter to take stereotaxic coordinates, make holes in the exoskeleton and insert the oxygen sensors without harming the animal. As illutstrated in Fig G, aluminium T slots (from MakerBeam, https://www.makerbeam.com/) were used for assembling the frame. A precision compass with spindle guide, spring-bow head and center wheel (Staedtler Mars® comfort 551) has been fixed to the aluminum frame and modified to allow the immobilization of the head by reducing the risk of damage.


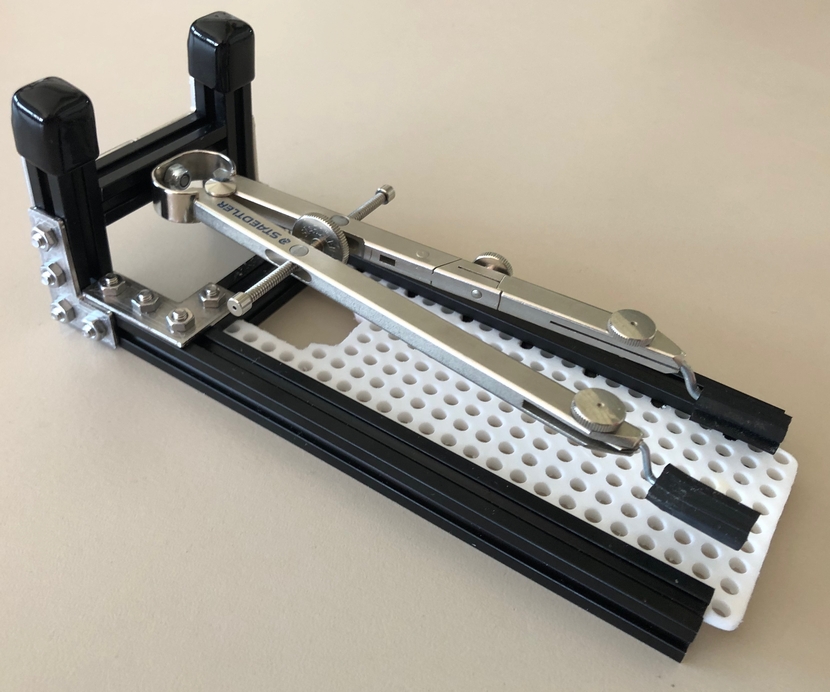


**Fig G. Stereotaxic frame adapter developed for immobilizing the head of the anesthetized animal during the neurosurgery.**

A sliding bed obtained by a plastic pegboard (<https://www.quercettistore.com>) was inserted in the frame for immobilizing the anesthetized cockroach with several rubber bands. A couple of rubber fixers, gently tightened on the head by mean of the compass center wheel and prevented harm to the animal during surgery (Fig H).


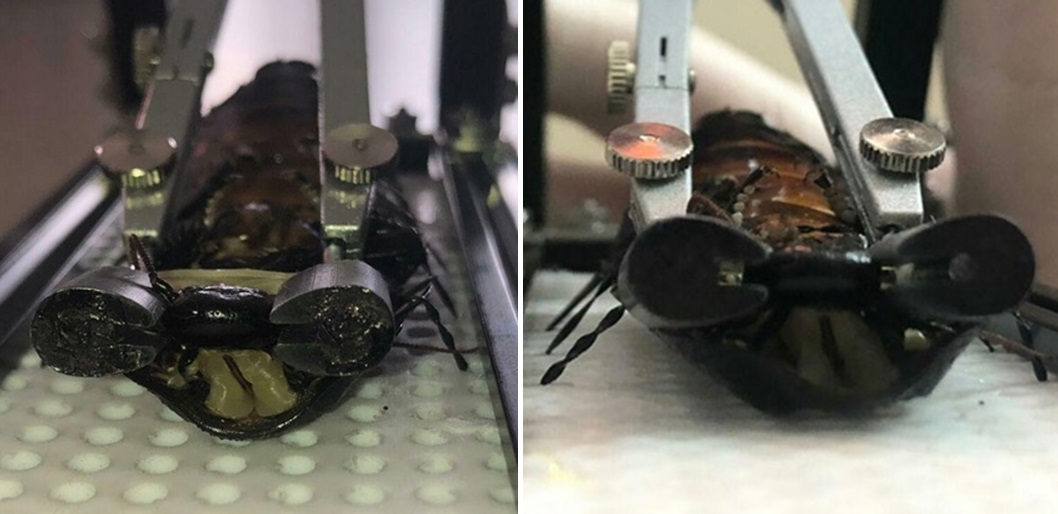


**Fig H. Anesthetized cockroach immobilized in the sliding bed with the head inside two rubber fixers.**

The adapter design allowed to quickly remove the animal from the frame by simply loosening the center wheel and sliding the bed. In this way it was possible to re-anesthetize the animal in a few seconds. Moreover, the holes of the pegboard facilitated the gas exchanges along the whole body of the cockroach. Once the head was immobilized, the adapter was fixed on the stereotaxic frame and the coordinates taken using the ocellar spots as reference points (Fig I).


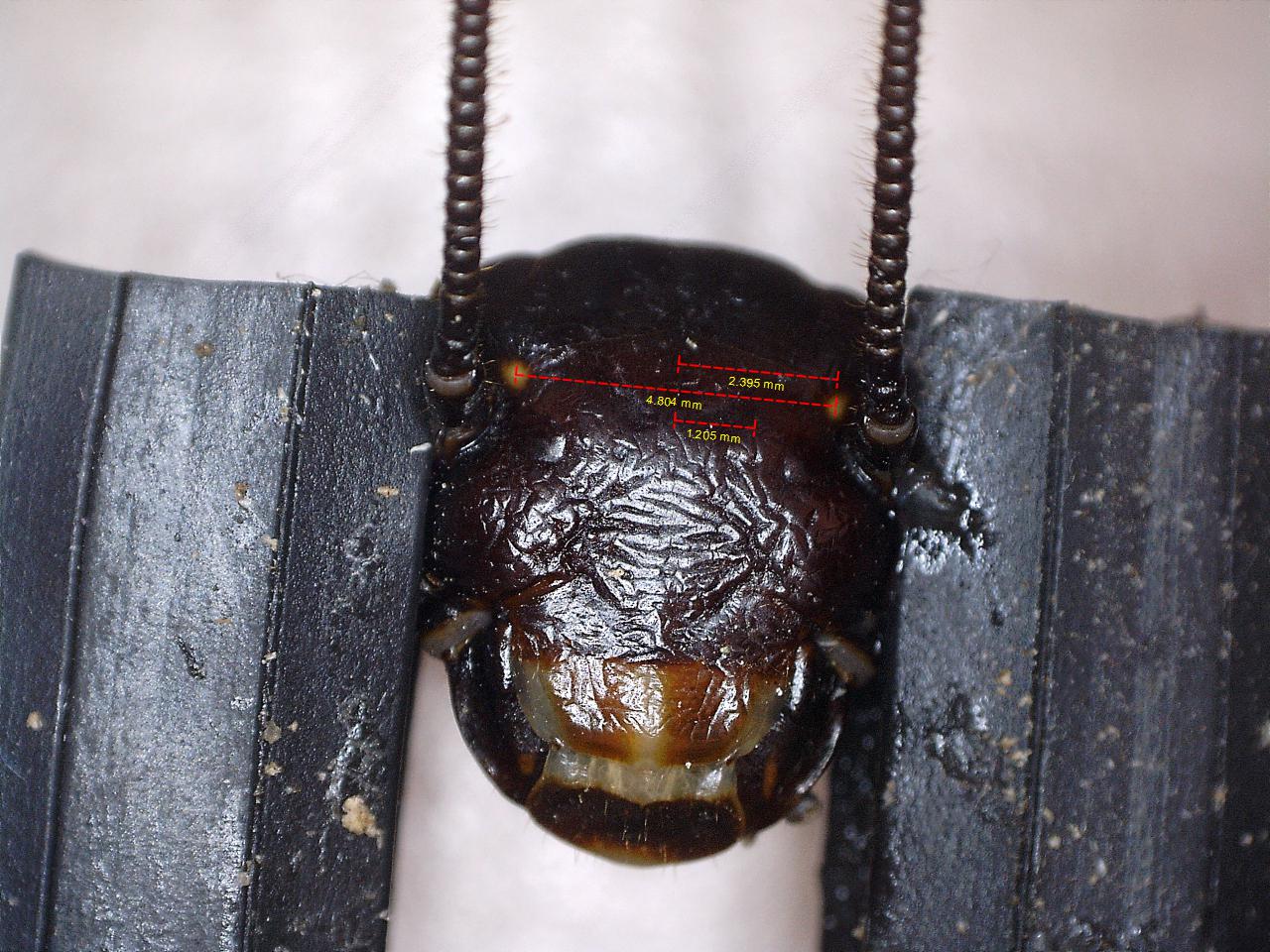


**Fig I. Microphotograph of the cockroach head and the measurements useful for taking the stereotaxic coordinates.**

**Stereotaxic surgery and *in vivo* experiments**

The stereotaxic surgery has been described in the main manuscript. Fig L illustrates several steps of the surgery starting from the realization of the holes with a spherical drill bit until the insertion of the screw and the consolidation of the implant through a UV-curing cement.


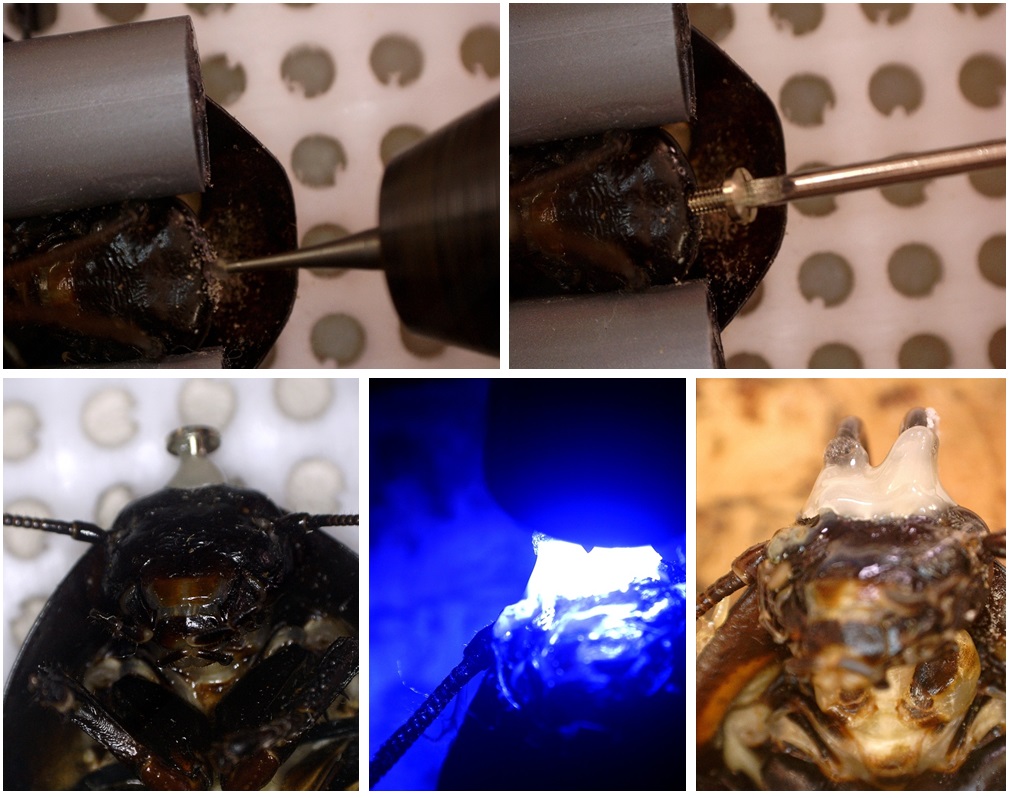


**Fig L. Selected steps of the stereotaxic surgery.**

One day after surgery, the implanted sensors were polarized by simply connecting the battery-powered telemetry device to the animal awake and free to move (Fig M).


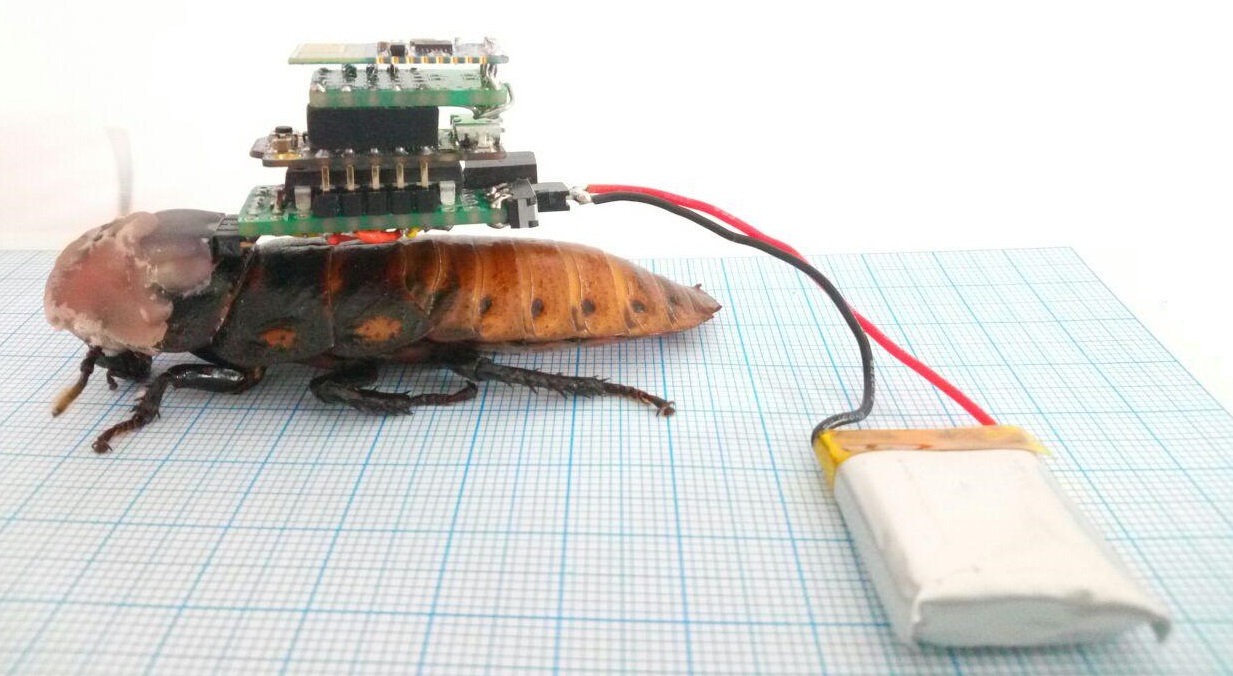


**Fig M. A freely-walking cockroach with its own telemetry device and the connected battery.**

**References**

1. Bazzu G, Puggioni GM, Dedola S, Calia G, Rocchitta G, Migheli R, et al. Real-time monitoring of brain tissue oxygen using a miniaturized biotelemetric device implanted in freely moving rats. Anal Chem. 2009;81: 2235–2241. doi:10.1021/ac802390f

2. Russell DM, Garry EM,Taberner AJ, Barrett CJ, Paton JFR, Budgett DM, et al. A fully implantable telemetry system for the chronic monitoring of brain tissue oxygen in freely moving rats. J. Neurosci. Methods. 2012;204:242-248. doi:10.1016/j.jneumeth.2011.11.019
