# Supplementary material for: Real-time telemetry monitoring of oxygen in the central complex of freely-walking Gromphadorhina portentosa
Source: PLoS One. 2019 Nov 11;14(11):e0224932. doi: 10.1371/journal.pone.0224932 (PMC6844484; doi:10.1371/journal.pone.0224932)
Supplement: Attachment — Submitted filename: Response to Reviewers.docx [file pone.0224932.s003.docx]

**Point-by-point response to the Reviewer's comments** **(Reviewer #1)**

I would like to thank the Reviewer for the time He/She spent in reviewing the manuscript and the suggestions that will help to improve the impact of the study.

Reviewer #1 comments and suggestions:

**1) *"[...] the author can include a comparison table with a few recent articles to show the advantages of oxygen monitoring in terms of stability, power consumption, and other parameters related to the oxygen detection in the telemetric system".***

Accepting the reviewer's suggestion we have identified (in recent literature) two telemetric oxygen detection systems similar to the one proposed in the present study and we compared them using a table. The first system is the one developed by our work group in 2009 (Bazzu et al., 2009) while the second was developed by Russel and coworkers in 2012. In order to provide the required details without weighing down the main manuscript, a paragraph entitled “Comparison among oxygen-detection telemetry systems” has been added to the Supporting Information.
